# Small Bowel Perforation as an Initial Manifestation of Immune Deficiency and Dysregulation-Associated Lymphoma in a Patient With Rheumatoid Arthritis: A Case Report

**DOI:** 10.7759/cureus.112996

**Published:** 2026-07-20

**Authors:** Chisa Shibata, Naoya Sasaki, Seiichiro Tada, Masato Maeda

**Affiliations:** 1 Gastroenterological Surgery, Shizuoka City Hospital, Shizuoka, JPN

**Keywords:** gastrointestinal perforation, idd-lpd and lymphomas, immunosuppressant medications, methotrexate, rheumatoid arthritis

## Abstract

Lymphoid proliferation and lymphomas associated with immune deficiency and dysregulation (IDD-LPD and lymphomas) are rare entities that occur in patients with autoimmune diseases, such as rheumatoid arthritis (RA) on immunosuppressant medications. Herein, we report a case of a 66-year-old woman diagnosed with IDD lymphoma after emergency laparotomy for ileal perforation. She had a history of RA, had been treated with antirheumatic drugs for 15 years, and was recently suspected of having gastric cancer with multiple liver metastases by her primary care physician. The patient was referred to our hospital because of severe abdominal pain. Computed tomography (CT) revealed peritonitis with intra-abdominal free air, and emergency laparotomy was performed. Intraoperative findings revealed multiple nodules in the ileum with two perforations, in addition to a gastric tumor and multiple liver tumors. Partial resection of the perforated ileum and anastomosis were performed. Histopathological examination of the resected ileum confirmed diffuse large B-cell lymphoma, leading to a diagnosis of IDD-lymphoma. Antirheumatic drugs were discontinued after diagnosis. Although the liver lesions shrank after discontinuation, Gallium-67 scintigraphy showed diffuse liver uptake. After careful discussion with hematologists, chemotherapy was administered (rituximab plus cyclophosphamide, doxorubicin, vincristine, and prednisone, six cycles). One year after chemotherapy, the patient remained in remission, and her RA symptoms were stable without antirheumatic drugs.

## Introduction

Methotrexate (MTX) and other immunosuppressant medications used to treat autoimmune diseases, such as rheumatoid arthritis (RA), can cause lymphoproliferative lesions in some patients [1,2]. This condition was formerly referred to as MTX-associated lymphoproliferative disorder (MTX-LPD), which is defined as lymphoid proliferations and lymphomas associated with immune deficiency and dysregulation (IDD-LPD and lymphomas), according to the World Health Organization classification in 2022 [3]. In most cases of IDD-LPD and lymphoma, discontinuation of immunosuppressive drugs is effective, leading to spontaneous regression; however, chemotherapy may be required in some cases [4]. About 40-50% of IDD-LPDs and lymphomas occur at extranodal sites, such as the gastrointestinal tract, skin, liver, lungs, or spine, and rarely require surgical treatment [5-7].

Herein, we present a case of IDD-lymphoma involving multiple extranodal lesions in the stomach, liver, and small intestine, identified following ileal perforation.

## Case presentation

**Figure 1:**
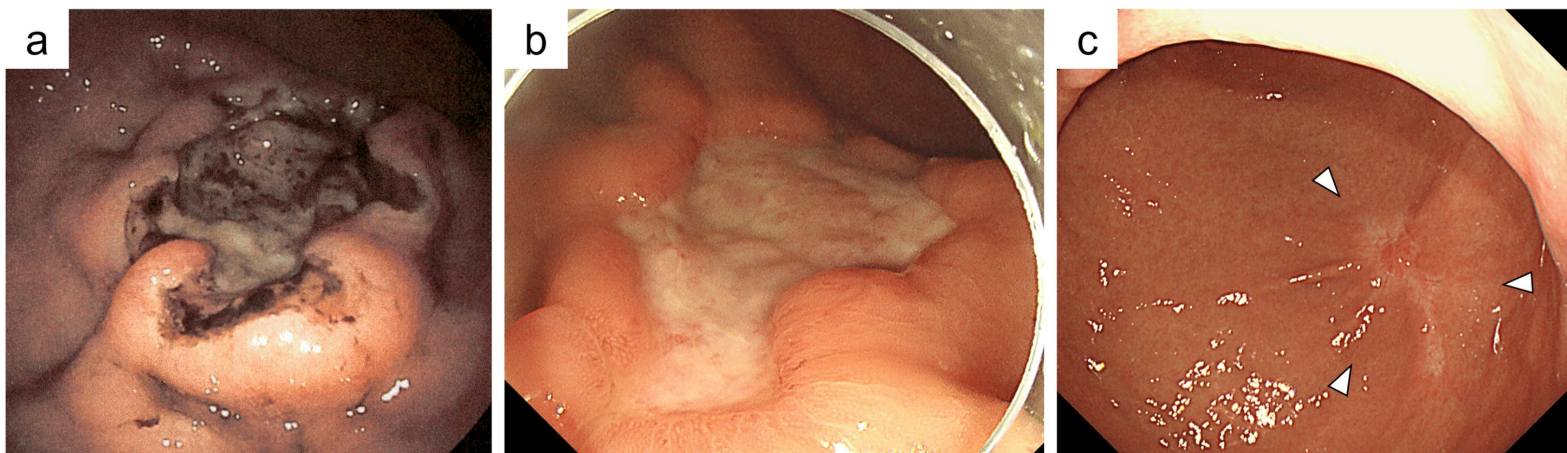
Esophagogastroduodenoscopy findings (a) Three days prior to emergency surgery, a Borrmann type III like tumor was detected on the greater curvature of the gastric body. (b) On postoperative day 11, the tumor had reduced in size, and the ulcer was healing. (c) Five months after chemotherapy, the ulcer had almost completely healed, with only a scar remaining.

A 66-year-old woman (163 cm, 86 kg, BMI 32.4 kg/m^2^) with severe general abdominal pain was referred to our hospital’s emergency department. She had a history of type 2 diabetes, hypertension, chronic heart failure, and a 15-year-history of RA treated with several immunosuppressant medications (MTX, iguratimod, tacrolimus hydrate, sulfasalazine, and baricitinib). She had received methotrexate at 8 mg/week for 15 years, iguratimod at 50 mg/day, tacrolimus at 1 mg/day, sulfasalazine at 1000 mg/day, and baricitinib at 2 mg/day. She also had chronic epigastric pain for several months before admission. Esophagogastroduodenoscopy (EGD) and abdominal ultrasonography performed at her primary care clinic revealed a tumor in the greater curvature of the gastric body (Figure 1a) and multiple mass lesions in the liver.

**Figure 2:**
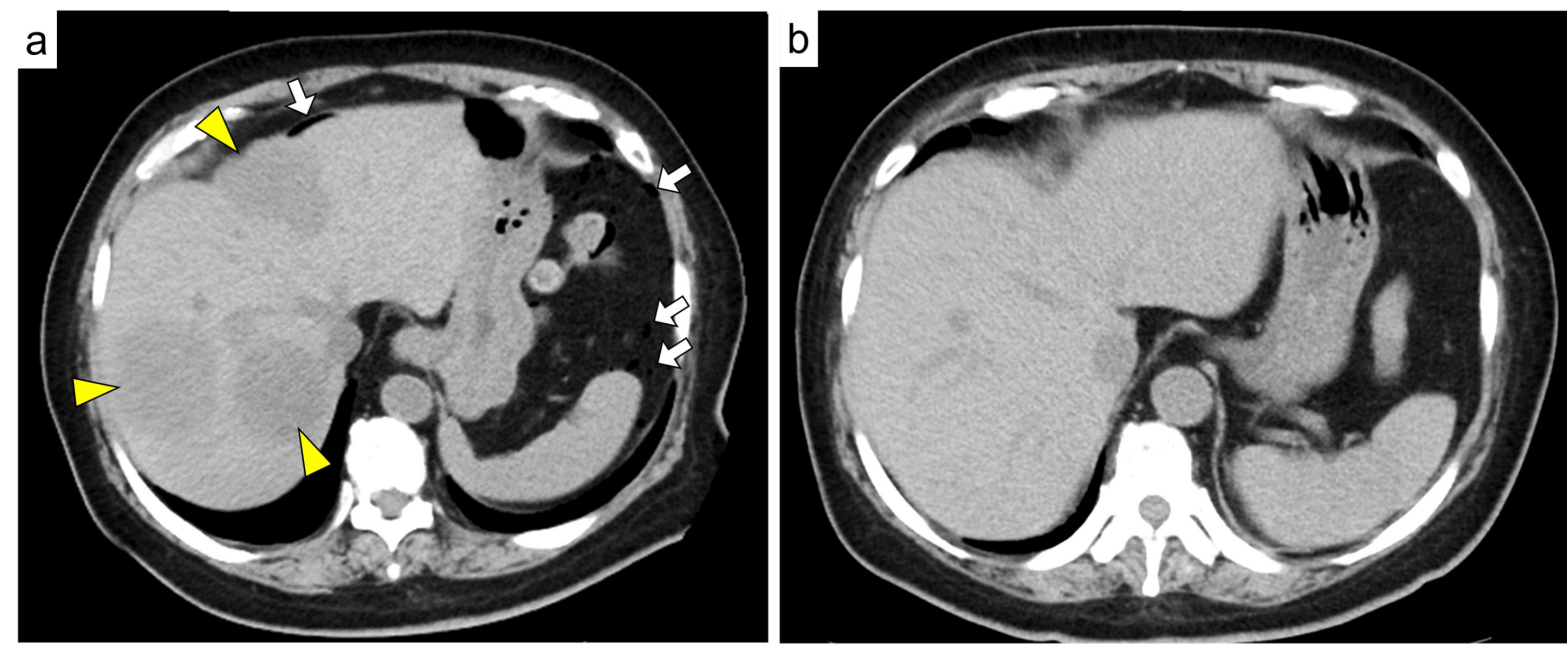
Abdominal computed tomography findings (a) Preoperative imaging revealed free air on the liver surface (arrows). The site of perforation was not identifiable. Multiple low-attenuation areas were observed in the liver (arrowheads). (b) On postoperative day 14, the low-attenuation areas in the liver had almost completely resolved.

**Figure 3:**
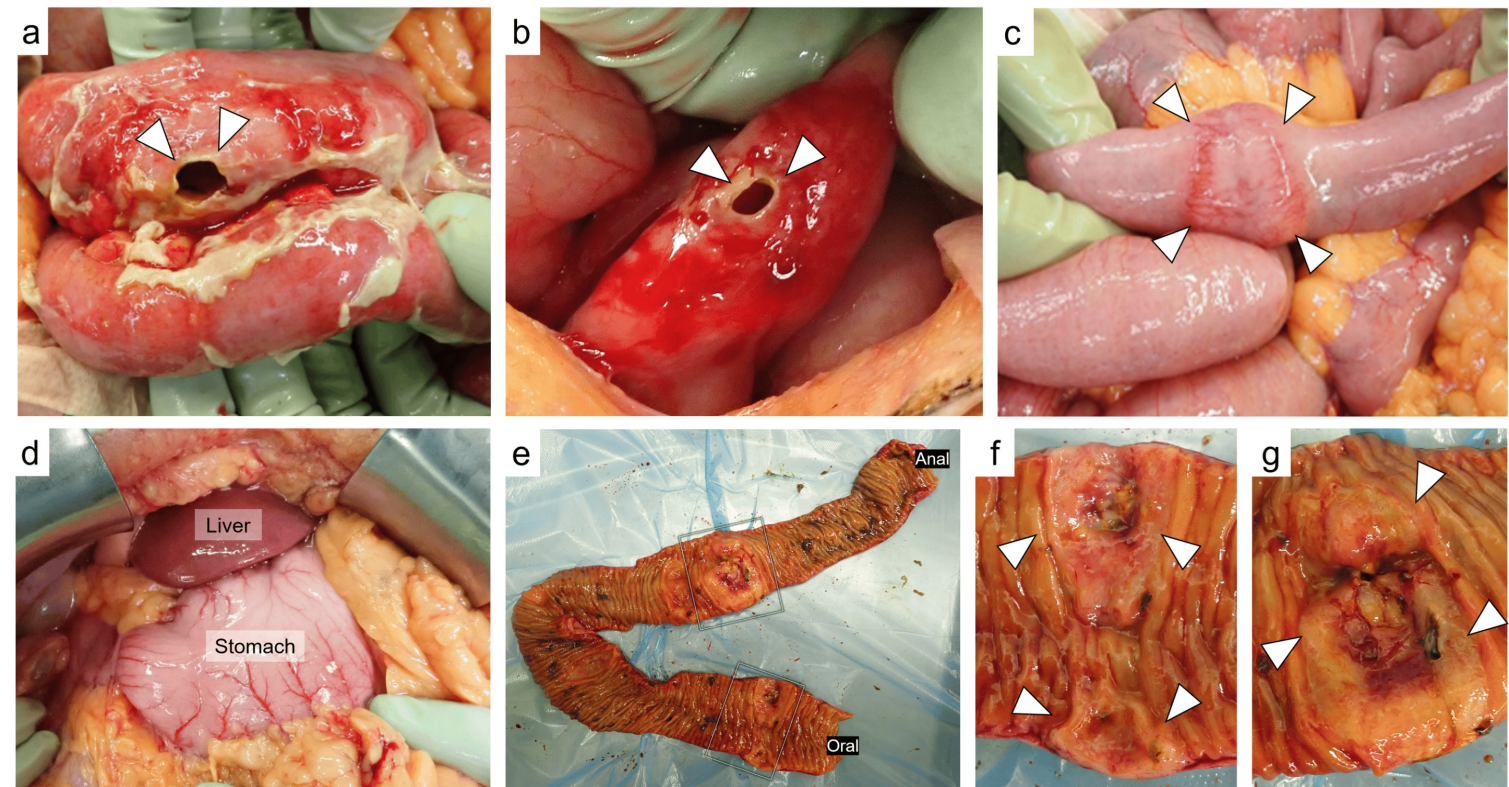
Intraoperative findings (a) Perforation on the antimesenteric side of the ileum, 160 cm from the ileocecal valve (arrowheads). (b) Perforation on the antimesenteric side of the ileum, 120 cm from the ileocecal valve (arrowheads). (c) Multiple intramural nodules throughout the small intestine (arrowheads). (d) No serosal exposure of the gastric or hepatic tumors. No peritoneal dissemination. (e) Resected specimen. (f) (g) Enlarged view of boxed area in panel e. Perforation at the base of the tumor ulcer (arrowheads).

On general examination, her blood pressure was recorded as 106/60 mmHg, pulse rate 117 beats/min and regular, body temperature 36.2°C, and peripheral oxygen saturation on room air as 96%. Abdominal examination showed moderate tenderness around the umbilicus but no obvious Blumberg’s sign. Laboratory test results demonstrated elevated inflammatory markers; white blood cell count of 10.7×103 /μL (normal value 3.5-8.0×103 /µL), C-reactive protein of 31 mg/dL (normal value < 0.3 mg/dL), moderate anemia; hemoglobin of 7.6 g/dL (normal value 11.5-15.0 g/dL), and the elevation of lactate dehydrogenase of 412 U/L (normal value 120-222 U/L). Preoperative laboratory findings are summarized in Table 1. Computed tomography (CT) revealed free air mainly in the upper abdominal cavity, thickening of the wall of the gastric body, edematous small bowel, and multiple low-attenuation areas of the liver (Figure 2a). Based on these findings, a gastrointestinal perforation, probably due to a gastric tumor, was diagnosed, and an emergency laparotomy was performed. Intraoperative findings showed 5 and 3 mm perforations approximately 120 and 160 cm proximal to the terminal ileum, respectively (Figures 3a, 3b). Multiple nodules were observed throughout the small bowel (Figure 3c). Multiple liver tumors and a tumorous lesion in the stomach were palpable, with no serosal invasion, and no obvious disseminated lesions were observed within the abdominal cavity (Figure 3d). Approximately 60 cm of the small intestine with perforated sites was resected (Figures 3e, 3f, 3g), and functional end-to-end anastomosis was performed.

**Table 1:** Preoperative laboratory data AST: aspartate aminotransferase, ALT: alanine aminotransferase, ALP: alkaline phosphatase, LDH: lactate dehydrogenase, GGT: gamma-glutamyl transferase, CRP: C-reactive protein, BUN: blood urea nitrogen, WBC: white blood cell count, RBC: red blood cell count, CEA: carcinoembryonic antigen, CA19-9: carbohydrate antigen 19-9

Test(Unit)	Result	Reference range
AST (U/L)	56	10-40
ALT (U/L)	39	5-45
ALP (U/L)	135	38-113
LDH (U/L)	412	120-222
GGT (U/L)	117	<30
Serum albumin (g/dL)	2.9	3.7-5.5
CRP (mg/dL)	31	<0.3
Total bilirubin (mg/dL)	0.6	0.2-1.2
BUN (mg/dL)	37.1	8.0-20.0
Creatinine (mg/dL)	1.14	0.47-0.79
WBC (×10^3^/μL)	10.7	3.5-8.0
RBC (×10^6^/μL)	2.28	3.8-4.8
Hemoglobin (g/dL)	7.6	11.5-15.0
Platelet (×10^3^/mL)	471	120-400
CEA (ng/mL)	0.9	<5.0
CA19-9 (U/mL)	7.9	<37.0

**Figure 4:**
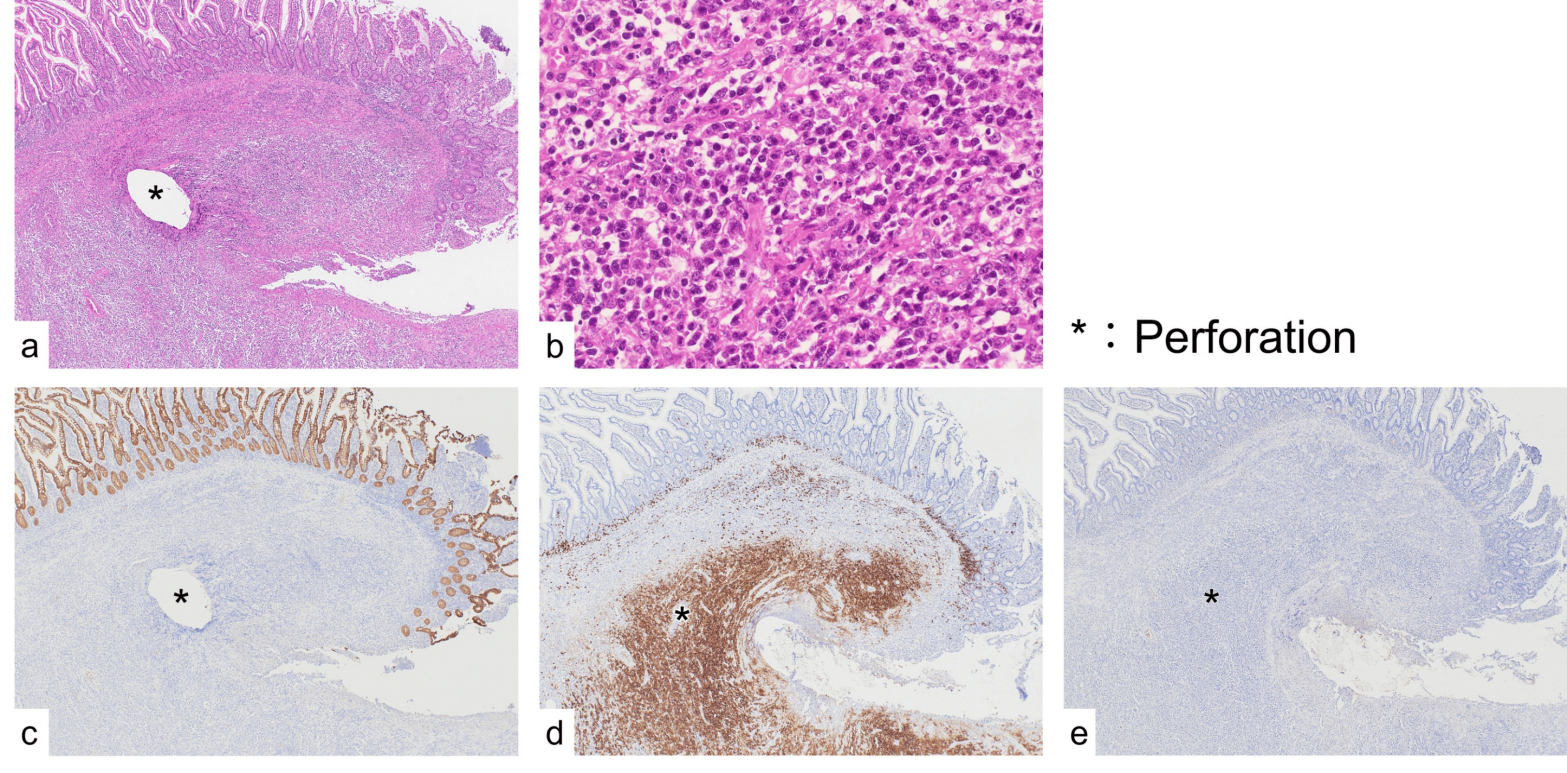
Microscopic findings (a) HE staining, low magnification (×40). Asterisk indicates tumor perforation site. (b) HE staining, high magnification (×400). (c) Cytokeratin (AE1/AE3) immunostaining. Negative. (d) CD20 immunostaining. CD20-positive tumor cells were observed at the perforation site. (e) Epstein-Barr virus-encoded RNA in situ hybridization. Negative.

**Figure 5:**
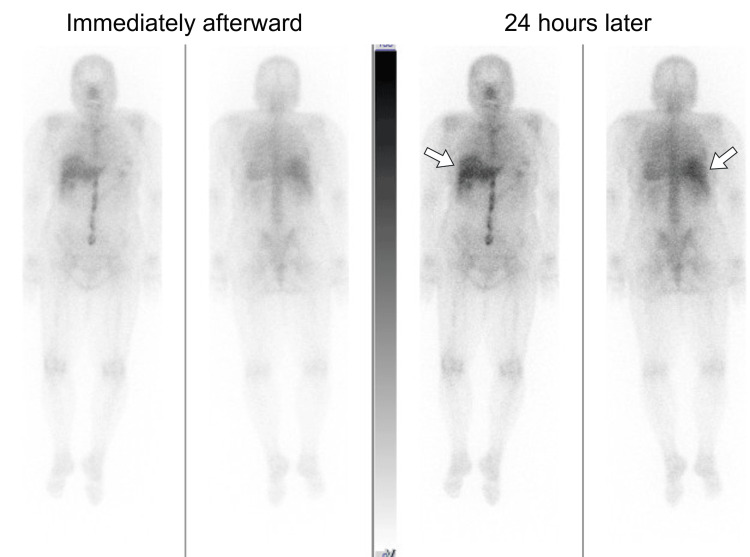
Gallium-67 scintigraphy Delayed imaging at 48 hours demonstrated faint hepatic uptake (arrows).

The patient began oral intake on postoperative day (POD) 1 and dietary intake on POD 3. As her condition was generally stable, all antirheumatic medications were resumed on POD 7. On POD 10, histopathological examination of the resected specimen confirmed diffuse large B-cell lymphoma (DLBCL) causing small bowel perforations (Figures 4a-4d). Epstein-Barr virus-encoded RNA (EBER) was negative (Figure 4e), and the postoperative serum soluble interleukin-2 receptor (sIL-2R) level was elevated to 900 U/mL (normal value 120-500 U/mL). On POD 11, progressive anemia and melena were observed; subsequent emergent EGD showed no obvious source of bleeding within the stomach, and the gastric tumor had reduced in size (Figure 1b). On the same day, a consultation with a hematologist suggested the possibility of IDD lymphoma, and all antirheumatic drugs were discontinued. Contrast-enhanced CT performed on POD 14 showed marked shrinkage of multiple liver masses (Figure 2b). In the following days, melena and anemia improved with conservative treatment. On POD 25, gallium-67 scintigraphy was performed for DLBCL staging, which revealed faint uptake in the liver (Figure 5). Since lymphoma involvement was identified in multiple extranodal organs, including the stomach, liver, and small intestine, the patient was classified as Stage IV DLBCL according to the Ann Arbor staging system, and then R-CHOP therapy was initiated on POD 30. After the completion of six cycles, 18F-fluorodeoxyglucose positron emission tomography showed no abnormal uptake. EGD also showed that the gastric mass had nearly disappeared (Figure 1c), and the sIL-2R level had reached the normal range. One year after chemotherapy, she remained well without DLBCL relapse, and the symptoms of RA were controlled without antirheumatic medications.

## Discussion

IDD-LPDs and lymphomas were initially reported in 1991 as LPD unrelated to RA activity in a patient treated with MTX [8]. Subsequently, following reporting of similar cases, the entity was classified as "MTX-LPD" in the 3rd edition (2001) of the WHO Classification. Since then, there have been several reports of LPD occurring during treatment with immunosuppressive agents other than MTX. Currently, all LPDs that arise during immunosuppressive therapy for autoimmune diseases or other inflammatory conditions, excluding post-transplantation settings, are classified in accordance with the revised 5th edition of the WHO classification as IDD-LPDs and lymphomas [3].

The diagnostic criteria for IDD-LPD and lymphoma are based on comprehensive histology, viral status, and clinical setting. The present case had an iatrogenic immunodeficiency background other than post-transplantation and was diagnosed with DLBCL based on a specimen of the perforated small bowel. In addition, discontinuation of several immunosuppressant medications led to spontaneous regression of gastric and hepatic lesions, which is one of the characteristic clinical features of patients with IDD-LPD and lymphoma [4].

The morphological presentation of lymphomas is mainly associated with the severity of immunodeficiency and the dysregulation of specific immune cell populations [9]. IDD-LPD and lymphoma patients are known to have a relatively high incidence of extranodal disease (40-50%) [10]. Among the diverse extranodal lesions in IDD-lymphomas, the head and neck region (including the oral and nasal cavities) accounts for 24%, the chest 15%, and the abdomen 14% [6]. In terms of gastrointestinal tract lesions, most previous cases were incidentally detected on CT or EGD or colonoscopy, whereas it is relatively rare for IDD-lymphomas to be diagnosed as a result of gastrointestinal perforation [9,11-15].

The histopathological features of IDD-LPDs and lymphomas are diverse. DLBCL accounts for approximately half of cases, followed by Hodgkin lymphoma at approximately 20% [6]. Among a certain proportion of these cases, the Epstein-Barr virus was detected by in situ hybridization with an EBER probe [16]. Although spontaneous regression can be expected in patients with IDD-LPD and lymphoma after discontinuation of immunosuppressant drugs, if EBER is negative, spontaneous regression is less likely than in EBER-positive cases [16-18]. For this reason, EBER status would be an important factor in proceeding with further treatment, such as chemotherapy, after the discontinuation of immunosuppressant drugs.

In the present case, the gastric and hepatic lesions were initially thought to be gastric cancer with liver metastases, which are more common in clinical settings. In particular, gastrointestinal surgeons would have more limited opportunities to manage IDD-lymphomas than hematologists and gastroenterologists because the main treatment for IDD-lymphomas is non-surgical. If we had recognized the possibility of IDD-lymphoma considering the patient’s immunosuppressive background and intraoperative findings, postoperative management, such as the earlier discontinuation of immunosuppressant medications, would have been optimal. Although IDD lymphomas are rarely encountered in the daily practice of gastrointestinal surgeons, it is important to consider them because early diagnosis can facilitate minimally invasive treatment and prompt consideration of postoperative chemotherapy.

## Conclusions

Here, we present a case of IDD-lymphoma that was diagnosed after small bowel perforation. Gastrointestinal surgeons would have limited experience with patients with autoimmune diseases treated with immunosuppressant drugs and would poorly recognize IDD-lymphomas. When treating gastrointestinal perforations in patients with autoimmune diseases using immunosuppressant drugs, it is important to consider treatment-related lymphoma as a potential diagnosis. This can lead to the early consideration of minimally invasive treatments or postoperative chemotherapy.
